# Prognostic Value of MR Imaging Texture Analysis in Brain Non-Small Cell Lung Cancer Oligo-Metastases Undergoing Stereotactic Irradiation

**DOI:** 10.7759/cureus.584

**Published:** 2016-04-25

**Authors:** Valerio Nardone, Paolo Tini, Michelangelo Biondi, Lucio Sebaste, Eleonora Vanzi, Gianmarco De Otto, Giovanni Rubino, Tommaso Carfagno, Giuseppe Battaglia, Pierpaolo Pastina, Alfonso Cerase, Lorenzo Nicola Mazzoni, Fabrizio Banci Buonamici, Luigi Pirtoli

**Affiliations:** 1 Unit of Radiation Oncology, University Hospital of Siena, Siena, Italy; 2 Department of Medical Physics, University Hospital of Siena, Siena, Italy; 3 Unit of Neuro Radiology, University Hospital of Siena, Siena, Italy

**Keywords:** texture analysis, stereotactic irradiation, brain metastases, srs, non single cells lung cancer

## Abstract

**Background:**

Stereotactic irradiation is widely used in brain oligo-metastases treatment. The aim of this study is to evaluate the prognostic value of magnetic resonance imaging (MRI) texture analysis (TA) of brain metastases (BM) of non-small cell lung cancer (NSCLC).

**Materials and methods:**

This study included thirty-eight consecutive patients undergoing stereotactic irradiation, that is, stereotactic fractionated radiotherapy (SRT) or radiosurgery (SRS), from January 2011 to December 2014 for 1-2 brain BM from NSCLC. Whole-brain radiotherapy (WBRT) was not delivered. The diagnostic MRI DICOM (Digital Imaging and Communications in Medicine) images were collected and analyzed with a homemade ImageJ macro, and typical TA parameters (mean, standard deviation, skewness, kurtosis, entropy, and uniformity) were evaluated for: brain progression-free survival; modality of brain metastatic progression (local progression or/and new metastases); and overall survival, after SRT/SRS.

**Results:**

After SRT/SRS 14 patients (36.8%) experienced recurrence in the brain, with a recurrence in the irradiated site (five patients, 13.2%), new metastases (11 patients, 28.9%), local recurrence and new metastases (two patients, 5.25%). Nineteen patients (50%) died of tumor progression or other causes. Entropy and uniformity were significantly associated with local progression, whereas kurtosis was significantly associated with both local progression and new brain metastases.

**Conclusions:**

These results appear promising, since the knowledge of factors correlated with the modality of brain progression after stereotactic irradiation of brain oligo-metastatic foci of NSCLC might help in driving the best treatment in these patients (association of SRT/SRS with WBRT? Increase of SRT/SRS dose?). Our preliminary data needs confirmation in large patient series.

## Introduction

The reported incidence of brain metastases (BM) varies widely from 20% to 50%, and primary lung cancer shows the highest rate (18%-65%) [1]. The incidence of BM is rising due to several factors, including the improved effectiveness of cancer therapy and the reliability of modern imaging, that anticipates the diagnosis of BM [2]. Among non-small cell lung cancer (NSCLC) histologic types, the frequency of BM is higher in adenocarcinoma, than in squamous cell carcinoma [3]. Contrast-enhanced MRI represents the gold standard imaging study for the diagnosis of BM [4]. A common localization is the junction of the grey with the white matter; circumscribed margins and vasogenic edema are typical features [5]. Without treatment, the median survival of patients is extremely poor (four to seven weeks) [6], whereas various therapeutic approaches may attain median survival values ranging between 3.02 and 14.78 months, but usually not exceeding seven months [7].

Improvements in imaging, radiotherapy (RT), neurosurgery, and systemic therapy (chemotherapy, molecular target agents, immunotherapy), have dramatically changed the management of advanced-stage NSCLC (including BM). Present BM treatment includes: stereotactic irradiation delivered in a fractionated course (stereotactic radiotherapy, SRT); or in a single session (stereotactic radiosurgery, SRT); whole-brain radiation therapy (WBRT); neurosurgical resection, or best supportive care; while chemotherapy, target therapy and immunotherapy are suitable for treatment of BM arising from particular primaries (e.g., melanoma) [8-10]. SRS and SRT are radiation therapy focal techniques, which in most cases can be performed with a linear accelerator, by using stereotactic coordinates and multiple, collimated, convergent beams of high-energy photons to deliver high radiation doses to well-defined targets, thus satisfactorily sparing normal tissues [11]. Current clinical literature recommends SRS/SRT treatment for one to four BM (usually in association with WBRT), and WBRT alone for multiple metastases [12-14], although presently SRS is sometimes used also for multiple BM [15-16], with WBRT potentially used as a salvage regimen [17]. SRT is usually preferred to SRS when the target is exceedingly large or close to critical brain structures [18]. Synchronous boost strategies with SRS/SRT and/or WBRT to treat multiple targets at different doses have been reported [19-20].

In this context, it is of crucial interest to select the optimal treatment strategy. Coping with BM is challenging due to a multitude of factors which may influence the therapeutic strategy. Therefore, easy-to-use and reliable criteria are warranted for a safe control of brain metastatic cancer to improve both expectancy and quality of life. MRI-based texture analysis (TA) may have such requisites. This method gives a quantitative measure of the imaging heterogeneity that the naked eye may not appreciate, possibly reflecting inherent characteristics of aggressiveness. Different methods can be applied, including the statistical model and transform-based methods, and they have been already used in the treatment of both primary and metastatic brain cancers [21-23]. In this study, we investigated the potential role of TA as a predictive factor of the pattern of further brain progression in patients with BM from NSCLC, after SRS/SRT.

## Materials and methods

### Patient series

Between January 2011 and December 2014, 38 patients with one to two BM (diameter ≤ 3 cm) from NSCLC were consecutively referred and submitted to linear-accelerator-based SRT/SRS as described below. They are the subject of this study. Clinical and pathological data were recorded at referral, as follows: primary histology, blood counts and chemistry; Karnofsky Performance Status (KPS) and a brain MRI scan before RT treatment. Patients with multiple BM (that is, > 3), KPS <70, or undergoing WBRT, and/or anti-angiogenic treatment were excluded from this evaluation. The study was authorized by our Institutional Review Board, and a signed informed consent was obtained from each patient for the anonymous use of the clinical data. All the followed procedures were in accordance with the ethical standards of the Helsinki Declaration (1964, amended most recently in 2008) of the World Medical Association.

### Texture analysis (TA)

**Figure 1:**
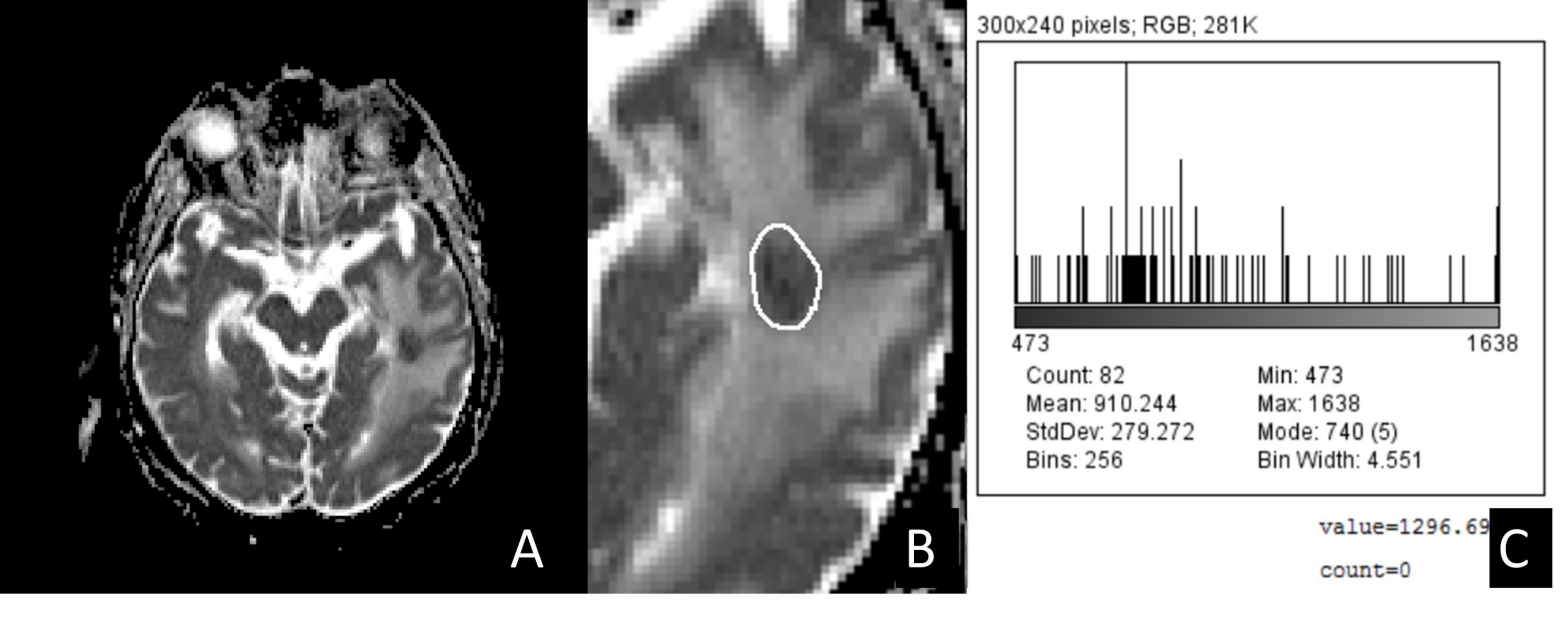
Contouring and Histogram of TA T2-MR Dicom image of a cohort patient (A), with a selected ROI (B) and a histogram of the TA (C).

The baseline MRI was performed according to a standard protocol, as follows: T1-, T2-, FLAIR- (Fluid Attenuated Inversion Recovery) acquisitions, with 1.25 mm slice thickness/1.5 mm separation; T1-gadolinium enhanced scans, in axial, coronal, and sagittal planes. We selected a region of interest (ROI); corresponding to the inner contour of each BM, using the T2 weighted sequence (see Figure 1).

**Figure 2:**
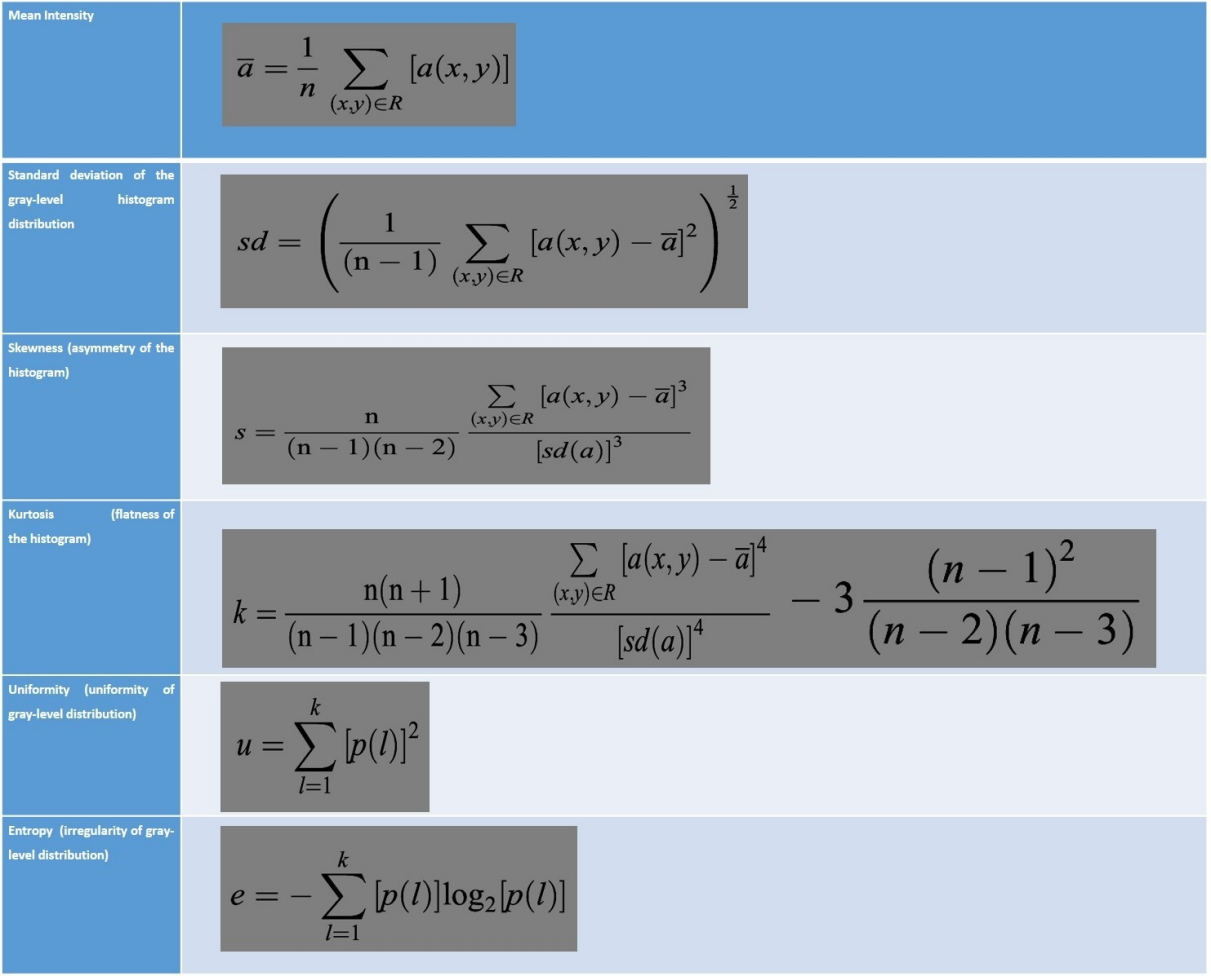
Mathematical Functions of TA Mathematical function implemented to calculate the TA parameters.

The ROI was contoured by a radiation oncologist (VN), in consensus with a neuroradiologist (AC) for RT planning. The TA was accomplished with a homemade ImageJ macro implementing a first order statistical-based method. This analysis can allow to take into account both distribution and relationships of the gray-level values quantitatively, that is, resulting in a gray-level frequency distribution from the histogram of the pixel intensities (Figure 1). We used an approach defined as the first order, in that it depended on single-pixel values rather than on the interaction with the neighboring pixels. We evaluated typical TA parameters, as follows: mean (m), standard deviation (sd), skewness s(sk), kurtosis (k), entropy (e), and uniformity (u). We report the mathematical function used to calculate these parameters in Figure 2.

In particular, mean and standard deviation refer to the mean intensity and to the standard deviation of the values in the histogram of pixel intensities, skewness refers to the asymmetry, and kurtosis refers to the flatness of the histogram. Uniformity and entropy are, respectively, parameters of the uniformity and of the irregularity of gray-level distribution. For each of the above parameters, two subgroups of patients (A, B) were identified, that is, below and above the median values respectively.

### Radiotherapy

RT was delivered with a Linear Accelerator 6 MeV photon beam, with a frameless stereotactic system. The treatment plan was accomplished with an XiO Treatment Planning System © (Elekta AB, Stockholm, Sweden), after target identification and contouring based on CT- MRI image fusions. The gadolinium-enhanced T1-MRI BM image identified gross tumor volume (GTV), and an isotropic margin (2-3 mm) was added to obtain the planning target volume (PTV). The SRS/SRT dose was prescribed to a reference isodose line, which was required to cover 100% of the GTV and more than 95% of the PTV. Coverage of 100% of the planning target volume by at least the prescription dose was required. The normal tissue constraints were met for all cases.

Radiosurgery (SRS) or hypofractionated RT (SRT) techniques were adopted on personalized bases, taking into account both tumor volume and proximity to critical structures, according to the Radiation Therapy Oncology Group (RTOG) criteria [24].

Twenty-eight patients were treated with SRS (73%) and 10 with SRT (27%). Median total dose for SRS treatment was 18 Gy (mean 17, 67 Gy, s.d. 3.5 Gy, range 14–23 Gy). Median total dose for SRT was 20 Gy (mean 20 Gy, s.d. 1.41 Gy, range 18–24 Gy, in 3-5 fractions).

### Follow-up

After completion of RT, all patients entered a scheduled follow-up program with brain MRI repetition at four and 12–16 weeks, then every six months or in any case showing clinical signs of progressive BM evolution (progressive disease, PD), in accordance with the Response Assessment in Neuro Oncology (RANO) criteria [25]. General and neurological examinations were obtained every three months.

End-points and statistical analysis are as follows. Primary end-point was overall survival (OS). Progression of BMs was recorded according to pattern of recurrence (PR) and time to progression (TTP), the former being analyzed as local progression of the treated BMs and/or as the onset of new BMs. We calculated TTP from the end of RT to the first evidence of PD at MRI, or by censoring follow-up data at the last available follow-up control. We estimated PR by analyzing MR images at PD. In order to differentiate local progression of the treated BMs from new BMs, we used the follow-up gadolinium-enhanced T1 sequences co-registered with the CT scans previously used for the RT treatment plan, by generating the original treatment plan, beam arrangement, and dose distribution again.

We analyzed survival values with the Kaplan-Meier method, according to the known survival predictive factors in NSCLC BMs (age, histology, KPS, number of metastasis, absence/ presence of extracranial metastases) and according to the TA parameters (m, sd, sk, k, e, u), assuming as cut-off values their respective median value.

## Results

### Characteristics of the patient series

The main characteristics of the patient series are summarized in Table 1.

**Table 1:** Characteristics of Patient Series

Factor of Interest	N	%
Sex		
Male	31	82%
Female	7	18%
Age		
>65	27	71%
≤65	11	29%
KPS		
<70	5	13%
70-80	25	65%
90-100	8	22%
Histology		
Adenocarcinoma	19	50%
SCC	17	45%
Unknown	2	5%
Extracranial metastases		
Present	26	68%
Absent	12	32%

Out of 38 patients, 31 (81%) were male and seven (19%) female. Histology of the primary lung cancer was found to be adenocarcinoma (AC) in 19 patients (50%), squamous cell carcinoma (SCC) in 17 patients (45%), and unknown lung cancer in two patients (5%). The median age was 72 years (mean 71 years, s.d. 8.51 years, range 52-87 years). KPS was < 70 in five patients (15%), 70-80 in 20 (60%), and > 80 in eight (25%). Only 10 patients (27%) did not show extra-cranial metastatic disease. Thirty patients had only one (78%) and eight patients two BMs (22%).

### Clinical outcome

The median OS was 3.5 months (mean 8.82 months, s.d. 15.37 months, range 1-92 months). The female sex was significantly associated with a lower OS (p= 0.008), a lower KPS score (p= 0.030) and the presence of extracranial metastases (p<0.001). During the follow-up (12-48 months), 14 patients (36%) showed evidence of BM progression, five patients (13%) showed evidence of local recurrences, 11 patients (29%) showed evidence of new BMs, and two patients (5.25%) showed evidence of both local and new BMs. Nineteen patients (50%) died of progressive NSCLC in brain or other sites, or of other causes. Median time to local progression (L-TTP) was 3.5 months (mean 8.97 months, s.d. 15.66 months, range 1-92 months), median time to new BM (N-TTP) was three months (mean 6.11 months, s.d. 6.34 months, range 1-26 months).

### Analysis of factors predicting time / modality of PD

Out of the known survival predictive factors in NSCLC BMs (age, KPS, number of BMs, absence / presence of extracranial metastases), only the presence of extracranial disease was significantly associated with time for new BMs progression (N-TTP, p= 0.004). Lower entropy (p= 0.086) and a higher uniformity (p= 0.086) show a correlation trend towards a poorer OS. The results are summarized in Table 2.

**Table 2:** Statistical Analysis of the L-TTP and N-TTP TA parameters are divided according to their respective median value (Subgroup A< Median value, Subgroup B> median value). * No statistics are computed because all cases are censored.

Factor of Interest	Mean of TTP	Standard Deviation	Significance
Age			
Local recurrence			0.632
>60	34.0	4.35	
≤60	9.0	2.45	
New metastases			0.179
>60	30.3	4.54	
≤60	8.7	2.84	
KPS			
Local recurrence			0.802
<70	*	*	
70-80	*	*	
90-100	*	*	
New metastases			0.731
<70	6.0	0	
70-80	13.24	2.54	
90-100	27.09	6.43	
Histology			
Local recurrence			0.238
AC	52.14	17.19	
SCC	17.25	1.73	
Distant metastases			0.111
AC	9.85	2.81	
SCC	15.11	2.04	
Extracranial metastases			
Local recurrence			0.279
Present	18.50	1.42	
Absent	30.18	5.52	
New metastases			0.024
Present	9.53	1.92	
Absent	34.27	6.13	
Sex			
Local recurrence			0.281
Male	*	*	
Female	*	*	
New metastases			0.005
Male	28.51	5.24	
Female	5.91	1.30	
Texture analysis			
Mean local recurrence			0.624
Subgroup A	20.85	3.07	
Subgroup B	15.84	2.04	
Mean new metastases			0.411
Subgroup A	17.17	3.83	
Subgroup B	10.39	2.16	
Standard deviation local recurrence			0.061
Subgroup A	14.85	3.67	
Subgroup B	18.60	1.32	
Standard deviation new metastases			0.344
Subgroup A	12.74	2.15	
Subgroup B	15.33	3.34	
Skew local recurrence			0.774
Subgroup A	21.80	2.65	
Subgroup B	15.25	2.17	
Skew new metastases			0.332
Subgroup A	11.86	3.09	
Subgroup B	13.37	2.59	
Kurtosis local recurrence			0.046
Subgroup A	22.40	17.93	
Subgroup B	8.39	6.19	
Kurtosis new metastases			0.023
Subgroup A	19.68	2.72	
Subgroup B	6.72	1.12	
Entropy local recurrence			0.013
Subgroup A	*	*	
Subgroup B	*	*	
Entropy new metastases			0.297
Subgroup A	10.88	3.29	
Subgroup B	14.11	2.40	
Uniformity local recurrence			0.013
Subgroup A	*	*	
Subgroup B	*	*	
Uniformity new metastases			0.297
Subgroup A	14.11	2.40	
Subgroup B	10.88	3.29	

The female sex was strongly associated with the pattern of recurrence (p= 0.004), as five out of seven (71.4%) female, vs. six out of 31 male patients (19.3%) developed new BM progression.

Histology was not associated with either OS, L-TTP or N-TTP.

Subgroups (A, B) subdivision for: m, sd, sk, k, e, and u are reported in Table 2 according to their median value. Entropy (e) showed a significant positive correlation with L-TTP: no statistics are computed, as all the cases over the median value are censored, with no in-field recurrence at the last follow-up control (p:0.013). Uniformity (u), instead, showed a significant negative correlation with L-TTP, and also in this case no statistics were computed as all the cases in the subgroup under the median value are censored, with no in-field recurrence at the last follow-up control (p:0.013). Kurtosis (k) was significantly associated with L-TTP, according to the same criteria as above (mean value: for subgroup A: 22.4 months, standard error 2.27 months, 95% CI 17-26 months; for subgroup B: 8.4 months, standard error 1.12 months, 95% CI 6-10 months, p:0.046). Kurtosis values for N-TTP were also significant (mean value: for subgroup A: mean 19.68 months, standard error 2.72 months, 95% CI 14-25 months; for subgroup B: 6.72 months, standard error 1.12 months, 95% CI 4-9 months, p:0.023).

**Figure 3:**
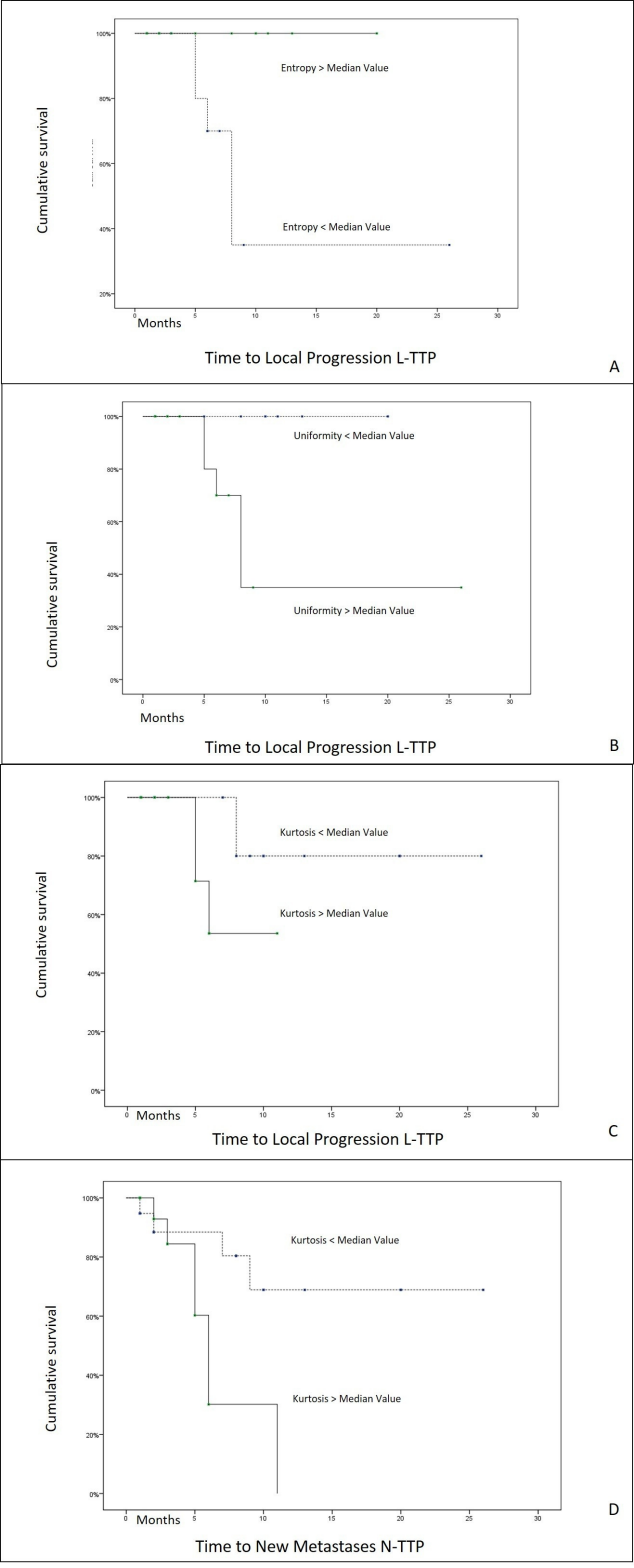
Kaplan-Meier Analysis Entropy (e) was significantly associated with L-TTP (no statistics are computed as all the cases in the subgroup over the median value are censored, with no in-field recurrence, p=0.013), as uniformity (u) (no statistics are computed as all the cases in the subgroup under the median value are censored, with no in-field recurrence, p=0.013) (A,B). Kurtosis (k) was significantly associated with L-TTP (mean value: for subgroup A: 22.4 months, standard error 2.27 months, 95% CI 17-26 months; for subgroup B: 8.4 months, standard error 1.12 months, 95% CI 6-10 months, p=0.046) and N-TTP (mean value: for subgroup A: mean 19.68 months, standard error 2.72 months, 95% CI 14-25 months; for subgroup B: 6.72 months, standard error 1.12 months, 95% CI 4-9 months, p=0.023) (C,D).

Kaplan Meier curves are shown in Figure 3.

## Discussion

Prognosis in NSCLC patients showing BM is extremely poor, as confirmed in the present series (3.5 months median OS) although many factors, such as age, Karnofsky performance status (KPS), number of BM, presence of extracranial metastases, account for different outcomes according to the Diagnosis-Specific Graded Prognostic Assessment (DS-GPA) score system [26]. In the related literature [27-29], not one of the different score systems devised for prognostic purpose in this patient category shows a clear superiority. However, choosing a correct therapeutic strategy in terms of effectiveness and toxicity is of the utmost importance, given the relevant proportion of NSCLC presenting BM during their clinical course. Stereotactic irradiation (SRS, SRT) of BM (associated or not with WBRT) is effective in this context [24]. A particular situation is the onset of a limited number of BM (1-4), in which SRS or SRT alone may represent a suitable treatment policy, with therapeutic results comparable to those achieved with aggressive RT or surgical strategies. In these patients, easy-to-use, practical, and reliable selection criteria for this kind of strategy are warranted. We attempted to investigate in this study a possible role for the MRI-based TA of BM in predicting the further evolution of the brain involvement after SRS / SRT. To this purpose, we retrospectively selected a particular sample of NSCLC patients, characterized by 1-2 BM of small size (≤ 3 cm); the majority of these patients showed adverse prognostic factors, such as advanced age (71% >65 y), poor polysaccharide-K (PSK) (78% <80), and extra-cranial metastases (68%). In such a clinical context, we believe that individuating parameters favoring an effective therapeutic approach with a limited aggressiveness, such as SRS/SRT alone is, could be important.

In the present experience, the MRI-based TA of BM has shown some promising data, to this regard. Through the quantitative measure of the image heterogeneity of BM allowed by this method, we attempted to investigate the possible inherent characteristics of aggressiveness of BM treated with SRS/SRT alone. We could show a significant positive correlation (p= 0.013) between time to local progression (L-TTP) and entropy of the irradiated BMs, reflecting in a better result for patients with e-values over the median (Figure 3A). A significant (p= 0.013) negative correlation of the same parameter (L-TTP) with Uniformity was shown, u-values under the median associated with a better outcome. The above L-TTP parameter, as well as time to new BM (N-TTP), were both significantly associated with Kurtosis (p= 0.046 and p= 0.023 respectively) and reflected better results in patients with k-value under the median.

However, it is difficult to infer how these parameters of image heterogeneity, detected by TA, may reflect inherent biological characteristics of NSCLC metastatic to the brain. The entropy and the uniformity was found to be significantly correlated with the L-TTP, so we can speculate that these parameters are an epiphenomenon of the radiosensibility and/or the vascularization of the brain metastases. The Kurtosis was found to be associated with both L-TTP and N-TTP. The lower flatness of the pixel distribution, consequently, could be related to the presence of an higher number of clonogenic cancer cells, thus representing an epiphenomenon of the aggressiveness of the primary tumor. These observations, anyway, should be interpreted cautiously and presently considered as empirical given also the lack of interpretative contributions by the related literature. TA, in fact, has already been used in brain tumors mostly for the differential diagnosis between radiation necrosis and brain metastasis [23], or to characterize different cancer metastases [22], or in pediatric brain tumors [30], and no specific and valuable data to this regard are available.

Some other criticisms to the present study can be raised. To date, many studies (including the present one) have used TA for limited tumor areas, e.g., the largest cross-sectional area, rather than for the whole tumor volume. Intra-tumor heterogeneity is likely to be greater over the whole tumor as compared to a limited region: hence, a cross-section TA may be inadequate and may impair reproducibility and the value of correlations between TA and clinical / pathobiological parameters.

The use of the same scanner and acquisition protocol for all the patients enrolled in the present study, together with the consensus in the definition of the region of interest (ROI) between a radiation therapist and a neuroradiologist, should partially overcome these limitations, without affecting the study conclusions. Anyway, the reproducibility of the TA needs to be tested in further studies.

Further, our analysis was accomplished with a homemade ImageJ macro, implementing a first order statistical-based method. With this analysis, it is possible to hold in account only the distribution and the relationships of gray-level values in a region of interest, thus considering the gray-level frequency distribution resulting from the histogram of pixel intensities. This is a first-order approach because it is dependent on single pixel values, rather than on interactions among neighboring pixels.

However, in our opinion the results of MRI-based TA for oligo-BM undergoing SRS/SRT reported here deserve consideration in that they might help to pave the way for a novel, image-based strategy in the clinical decision process for the considered NSCLC patient category. A systematic, prospective approach is mandatory for further research on this subject, grounded on large patient series and suitable databases including exhaustive clinical and pathobiological information.

## Conclusions

The results of MRI-based TA for oligo-BM undergoing SRS/SRT reported here deserve consideration because they might help to pave the way for a novel, image-based strategy in the clinical decision process for the considered NSCLC patient category. A systematic, prospective approach is mandatory for further research on this subject, grounded on large patient series and suitable databases including exhaustive clinical and pathobiological information.
